# Unveiling the hidden ally: *Blastocystis* links healthier diets and cardiometabolic benefits

**DOI:** 10.1038/s41392-025-02146-6

**Published:** 2025-03-03

**Authors:** Lei Deng, Kevin S. W. Tan

**Affiliations:** 1 Broad Institute of MIT and Harvard, Cambridge, MA USA; 2 Center for Computational and Integrative Biology, Massachusetts General Hospital, Boston, MA USA; 3 Laboratory of Molecular and Cellular Parasitology, Healthy Longevity Translational Research Programme and Department of Microbiology and Immunology, Yong Loo Lin School of Medicine, National University of Singapore, Singapore, Singapore

**Keywords:** Microbiology, Cardiology

In a recent study published in *Cell*,^1^ Piperni and colleagues revealed that *Blastocystis* prevalence varies significantly across geographic regions and lifestyles, with higher carriage linked to healthier plant-based diets and favorable cardiometabolic profiles. These findings position *Blastocystis* as a potential biomarker for gut health and metabolic well-being, challenging its traditional perception as a pathogen (Fig. 1).

**Fig. 1:**
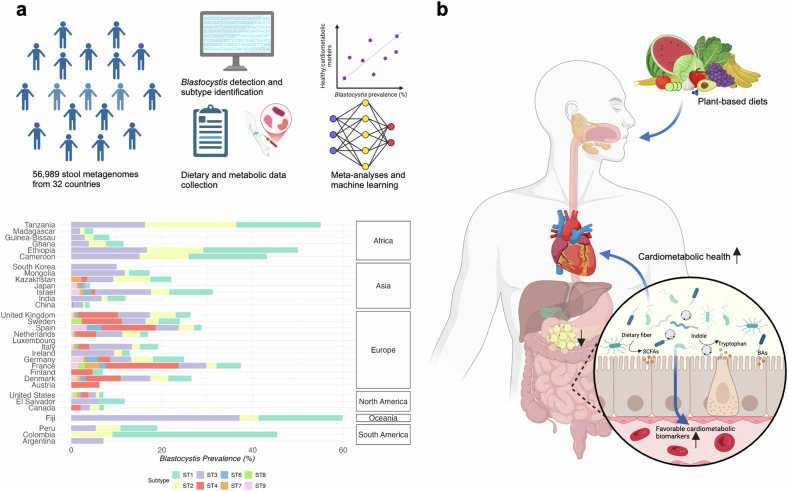
Global prevalence, dietary associations, and potential health implications of *Blastocystis*. **a** The analysis included metagenomic sequencing of stool samples from 56,989 individuals across 32 countries, accompanied by dietary and metabolic metadata. High-throughput sequencing and machine learning models (random forest) were employed to predict *Blastocystis* presence. The bar chart illustrates the geographic variability in *Blastocystis* prevalence (adapted from Piperni et al.). Fiji exhibited the highest prevalence (56.29%), while Japan had the lowest (2.46%). Subtypes (STs) varied by region, with ST1 and ST2 dominating in non-Westernized populations and ST4 more common in Europe. **b** The association of *Blastocystis* carriage with plant-based foods promotes gut health and correlates with increased *Blastocystis* abundance. It highlights the potential effects of *Blastocystis* on gut microbiome diversity, microbial metabolite production (e.g., SCFAs, BAs, tryptophan derivatives), and intestinal barrier integrity, which collectively contribute to favorable cardiometabolic biomarkers. Created with Biorender.com

The influence of diet on human health has garnered widespread attention in recent years, particularly in the prevention and management of cardiometabolic diseases. Research has shown that the gut microbiome plays a pivotal role in mediating the relationship between diet and health. While significant progress has been made in understanding gut bacteria, much less is known about the role of micro-eukaryotes such as *Blastocystis*. *Blastocystis* is a prevalent and genetically diverse gut microbe that, despite being identified over a century ago, has only recently been recognized for its potential contributions to host intestinal health.^2^ Traditionally regarded as a pathogen, emerging evidence now highlights *Blastocystis* interactions with the gut microbiome and its potential as a commensal or even a beneficial modulator of gut health. These findings signal a paradigm shift in *Blastocystis* research, shifting the focus from its presumed pathogenicity to its role in promoting gut microbial balance and overall health.

A comprehensive global analysis of stool metagenomes from 56,989 individuals across 32 countries was conducted, utilizing MetaPhlAn 4 for bacterial and archaeal classification and a machine learning model (random forest) to predict the presence of *Blastocystis*. This extensive survey revealed significant variability in global *Blastocystis* prevalence, ranging from 56.29% in Fiji to 2.46% in Japan. Given the remarkable genetic diversity within *Blastocystis*, distinct subtypes (STs) were found to have differential effects on the gut microbiota and host health. Further analyses revealed that *Blastocystis* ST carriage is influenced by geography and lifestyle. ST1 and ST2 were more common in non-Westernized populations, while ST4 predominated in Europe. These findings highlight the global variability in *Blastocystis* prevalence and underscore the influence of geography and lifestyle (Fig. 1a).

Dietary patterns profoundly shape the gut microbiome, influencing its composition and functional diversity. Piperni et al. demonstrated that individuals with higher *Blastocystis* carriage were more likely to consume plant-based foods such as dried fruits and vegetables—well-known for their gut health-promoting properties. *Blastocystis* presence was also associated with favorable cardiometabolic biomarkers, including lower diastolic blood pressure, C-peptide levels, triglyceride levels, and GlycA values, all of which are established risk factors for type 2 diabetes and cardiovascular disease. The study further verified that the effects of *Blastocystis* on cardiometabolic health were consistent across its subtypes, emphasizing its potential as a protective factor against chronic diseases like diabetes and heart disease (Fig. 1b).

Body adiposity, a key determinant of cardiometabolic health, is often assessed using body mass index (BMI) as a practical proxy. Individuals with higher *Blastocystis* prevalence consistently exhibited significantly lower BMI values. This association, along with favorable glucose homeostasis and reduced estimated visceral fat, was also observed in another large cohort study, further indicating a potential link between *Blastocystis* carriage and reduced body adiposity.^3^ Increased bacterial diversity is widely recognized as a hallmark of a resilient gut ecosystem, contributing to resistance against pathogenic colonization and overall microbial balance. Individuals harboring *Blastocystis* exhibited significantly higher alpha diversity in their gut microbiomes. Using random forest machine learning models, the study identified distinct microbial configurations associated with *Blastocystis* carriage. Specifically, *Blastocystis*-positive individuals showed higher relative abundances of beneficial bacteria such as *Ruminococcaceae*, while *Blastocystis*-negative samples were enriched with species like *Blautia* and *Bacteroides ovatus*. These findings suggest a robust ecological link between *Blastocystis* presence, enhanced gut microbiome diversity, and improved cardiometabolic profiles.

The study also highlighted significant increases in *Blastocystis* prevalence and abundance following dietary improvement interventions. In a personalized dietary intervention study involving 1124 healthy individuals, participants significantly improved their diet quality over a six-month period, leading to a marked increase in *Blastocystis* prevalence and abundance. These findings further support the positive correlation between *Blastocystis* carriage, healthy dietary habits, and cardiometabolic health.

The widespread presence of *Blastocystis* in healthy individuals and its positive association with plant-based diets and cardiometabolic health suggest a non-pathogenic or potentially beneficial role for this microbe in the gut. However, the mechanistic links between *Blastocystis*, dietary patterns, and cardiometabolic outcomes remain unclear. Short-chain fatty acids (SCFAs), produced by gut microbial fermentation of dietary fiber, are well-known for their cardiometabolic benefits. Notably, *Blastocystis* colonization has been shown to increase SCFA-producing bacteria and elevate SCFA concentrations.^4^ Additionally, the tryptophan metabolite indole-3-acetaldehyde (I3AA), produced by *Blastocystis*, has been found to regulate the balance between Treg and Th17 cells—immune cells that are also key regulators of cardiometabolic health.^5^ Further research is needed to explore how *Blastocystis* influences gut microbial metabolites, such as SCFAs, tryptophan, and bile acids (BAs), as well as its effects on intestinal barrier integrity and mucosal immunity.

This study highlights important clinical implications, particularly in dietary guidance and microbiome-based interventions. The link between *Blastocystis* carriage and healthier plant-based diets suggests that increasing fiber-rich food consumption could enhance gut microbial diversity and cardiometabolic health. Additionally, the potential role of *Blastocystis* as a biomarker of gut health opens avenues for its use in personalized nutrition strategies and microbiome-targeted therapies. For instance, microbiome profiling could integrate *Blastocystis* as an indicator of dietary quality or metabolic health, informing tailored dietary or therapeutic recommendations.

The study provides significant insights but also has certain limitations. First, the study relies on observational data, and the causal relationship between *Blastocystis* and cardiometabolic outcomes remains unclear. Future research should focus on controlled interventional studies and longitudinal analyses to establish causality and investigate how *Blastocystis* influences host metabolic health at the molecular level, with a particular focus on subtype-specific effects. Additionally, while increased bacterial diversity was observed in *Blastocystis*-positive individuals, the study does not explore the ecological interactions between *Blastocystis* and other microbial groups. Further investigation is needed to clarify the potential synergistic or competitive relationships between *Blastocystis* and other members of the gut microbiota. Lastly, it remains unclear whether *Blastocystis* directly contributes to health benefits or simply reflects a generally healthy lifestyle, warranting the development of in vitro and in vivo models to assess the functional roles of different *Blastocystis* subtypes and evaluate its potential as a therapeutic target.

In conclusion, this study leveraged an unprecedented metagenomic dataset spanning diverse geographic regions and applied machine learning techniques to uncover novel insights into the associations between *Blastocystis*, healthier dietary patterns, and improved cardiometabolic profiles. These results establish a strong foundation for exploring the genetic, genomic, and functional roles of *Blastocystis*, as well as its potential applications in personalized nutrition and microbiome-targeted therapies.

## References

[1] Piperni, E. et al. Intestinal *Blastocystis* is linked to healthier diets and more favorable cardiometabolic outcomes in 56,989 individuals from 32 countries. *Cell* **187**, 4554–4570.e18 (2024). 38981480 10.1016/j.cell.2024.06.018

[2] Tomiak, J. & Stensvold, C. R. Accelerating the paradigm shift in *Blastocystis* research. *Trends Parasitol* **40**, 775–776 (2024). 39069433 10.1016/j.pt.2024.07.006

[3] Asnicar, F. et al. Microbiome connections with host metabolism and habitual diet from 1,098 deeply phenotyped individuals. *Nat Med.* **27**, 321–332 (2021). 33432175 10.1038/s41591-020-01183-8 PMC8353542

[4] Deng, L. et al. Colonization with ubiquitous protist *Blastocystis* ST1 ameliorates DSS-induced colitis and promotes beneficial microbiota and immune outcomes. *NPJ Biofilms Microbiomes* **9**, 22 (2023). 37185924 10.1038/s41522-023-00389-1 PMC10130167

[5] Wojciech, L. et al. A tryptophan metabolite made by a gut microbiome eukaryote induces pro-inflammatory T cells. *EMBO J* **42**, e112963 (2023). 37743772 10.15252/embj.2022112963 PMC10620759

